# Formulation and Evaluation of Aceclofenac Injection Made by Mixed Hydrotropic Solubilization Technique

**Published:** 2010

**Authors:** Rajesh Kumar Maheshwari, Arpna Indurkhya

**Affiliations:** *Department of Pharmacy, Shri Govindram Seksaria Institute of Technology and Science, Indore, India.*

**Keywords:** Mixed-hydrotropy, Solubilization, Aceclofenac, Aqueous injection, Urea, Sodium citrate, Synergistic enhancement

## Abstract

Aceclofenac is a non-steroidal anti-inflammatory drug (NSAID) that exhibits analgesic, antipyretic and anti-inflammatory activities. It is practically insoluble in water. The effect of hydrotropes such as urea and sodium citrate and blends (urea + sodium citrate) on the solubility of aceclofenac was investigated. The enhancement in the solubility of aceclofenac was more than 5 and 25 folds in 30% sodium citrate solution and 30% urea solution, respectively, as compared to its solubility in distilled water. The enhancement in the solubility of aceclofenac in a mixed hydrotropic solution containing ≥ 20% urea and 10% sodium citrate solution was more than 250 folds (compared to its solubility in distilled water). This proved a synergistic enhancement in solubility of a poorly water- soluble drug due to mixed hydrotropy. Synergistic combination of hydrotropic agents can minimize the amount of hydrotropic agents employed, minimizing the chances of their toxicities. Aqueous injection of aceclofenac, using the mixed hydrotropic solubilization technique, was developed and by using the lyophilization method, the problem of inadequate stability of aceclofenac in aqueous solution was overcome. The developed formulation was studied for physical and chemical stability.

## Introduction

Hydrotropy is the term originally put forward by Neuberg (1) to describe the increase in the solubility of a solute by the addition of fairly high concentrations of alkali metal salts of various organic acids. However, the term has been used in the literature to designate non-micelle-forming substances, either liquids or solids, organic or inorganic, capable of solubilizing insoluble compounds. Hydrotropic solubilization process involves cooperative intermolecular interaction with several balancing molecular forces, rather than either a specific complexation event or a process dominated by a medium effect, such as co-solvency or salting-in. Hydrotropic agents have been observed to enhance the aqueous solubility of poorly water-soluble drugs (2-21).

Maheshwari has demonstrated the synergistic solubilizing capability due to mixed-hydrotropy approach and this approach has been applied to analyze the poorly water-soluble drug, aceclofenac, titrimetrically, precluding the use of organic solvents (2). He has nicely applied the application of hydrotropy in titrimetric and spectrophotometric estimations of a large number of poorly water-soluble drugs precluding the use of organic solvents (2-14). 

Mixed hydrotropic solubilization technique is the phenomenon to increase the solubility of poorly water-soluble drugs, using blends of hydrotropic agents (22). This technique can provide additive or synergistic enhancement effect on solubility of poorly water-soluble drugs. Utilization of this method in the formulation of dosage forms made of water insoluble drugs can also reduce the concentration of individual hydrotropic agents, in order to minimize the side effects (in place of using a large concentration of one hydrotrope, a blend of several hydrotropes can be employed in much smaller concentrations, reducing their individual toxicities). Hydrotropic solubilization technique for the formulation development of aqueous injection of various poorly water-soluble drugs has also been demonstrated (23-27). 

**Figure 1:**
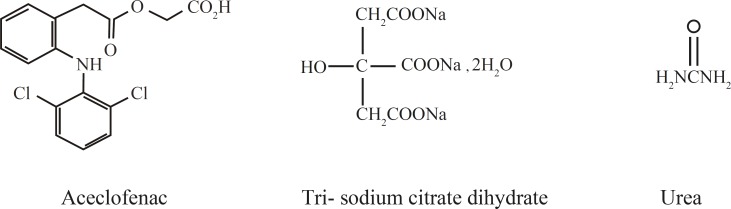
Chemical structure of aceclofenac and various hydrotropes

The objective of the present study is to explore the application of mixed hydrotropic solubilization technique in the formulation of dosage forms of water- insoluble/poorly water-soluble drugs and to reduce the concentration of individual hydrotropic agents to minimize their side effects. In the present work, aceclofenac, a non-steroidal anti-inflammatory agent was selected as a model drug which is a BCS class II drug (highly permeable and low soluble) and attempts were made to formulate an aqueous injection of this drug, using various hydrotropic agents. The formulation was also studied for physical and chemical stability. The chemical structure of drug and various hydrotropes used in this study are shown in Figure 1. 

## Experimental

*Materials*

The gift sample of aceclofenac was provided by IPCA Laboratories Ltd. Ratlam, India. Urea (Merck, Ltd., Mumbai, India) and tri-sodium citrate dihydrate (Loba chemie, Mumbai, India) were also used. All other chemicals and solvents used were of analytical/HPLC grade. Membrane filter (0.22 μm), (Sartorious, Germany), aluminium seal, glass vials (15 mL), and rubber plugs (Modern Labs, Indore, India) were also employed in this study. 

*Selection of hydrotropic blends*

It is evident from the literature survey that increasing the concentration of hydrotrope could increase the aqueous solubility of poorly water-soluble drugs. Therefore, highly concentrated solutions of hydrotropic agents were used in the present investigation. Distilled water was used in making hydrotropic solutions. 

As evident from a previous study (22), it was found that there was a significant enhancement in aqueous solubility (a synergistic effect) of aceclofenac by the use of a blend of urea (22.5%) and sodium citrate (22.5%). Keeping this point in mind and using a total dissolved hydrotrope concentration of at least 30%, different blends (Table 1) were made and the solubility of aceclofenac was determined in them.

**Table 1:** Hydrotropic blends selected for aceclofenac

**Blends **	**Urea **	**Sodium citrate **
A	15%	15%
B	20%	10%
C	20%	20%
D	25%	25%
E	30%	10%
F	30%	15%
G	30%	20%

*Determination of equilibrium solubility*

For equilibrium solubility determination at room temperature, the excess solute method was employed. Sufficient excess amounts of drug were added to screw capped 10 mL glass vials containing distilled water, solutions of individual hydrotropic agents, solution containing blends of hydrotropic agents and buffers of pH 7.4, 8.0 and 9.0 (pH range of hydrotropic solutions), separately. The vials were shaken mechanically for 12 h at room temperature in an orbital flask shaker (Khera Instruments Pvt. Limited, Delhi, India). The solutions were allowed to equilibrate for the next 24 h and then transferred into Eppendorf tubes and centrifuged for 30 min at 2000 rpm (Remi Instruments Limited, Mumbai, India). The supernatant of each vial was filtered through Whatman filter paper. Filtrates of saturated solutions of aceclofenac were analyzed by spectrophotmetric method using a double beam UV-visible spectrophotometer (Shimadzu^®^160A), measuring the absorbance of appropriately diluted solutions against the respective reagent blanks at 275 nm. Enhancement ratio in solubility was determined by the following formula:

Enhancement ratio=Solubility of drug in hydrotropic/Solubility of drug in distilled water 

*UV spectral studies*

In order to check any interaction between drug and the hydrotropic agents, UV spectral studies of aceclofenac were performed in different hydrotropic solutions. Possible spectroscopic changes in the structure of aceclofenac in the presence of hydrotropes were subsequently investigated.

*Thin layer chromatographic studies*

In order to examine the possibility of interaction between drug and hydrotropes, thin layer chromatographic studies were performed. A silica gel G 254 plate was activated at 110°C for 1 h. The methanolic solution of aceclofenac alone, the aqueous solution of hydrotropic solution, as well as solubilized product of aceclofenac in hydrotropic blend B (20% urea + 10% sodium citrate) solution were spotted on the base line with the aid of a microdropper. Then, the plate was left for 10 min to be dried and transferred to a solvent jar saturated with solvent system composed of mixture of chloroform, methanol and ammonia solution (48:11.5: 0.5 v/v/v) (28). 

The solvent system was allowed to run for a height of about 4 cm. Finally, the plate was transferred to an oven maintained at a temperature of 80°C for 5 min and then it was observed under UV light for visualization of spots. 

*Formulation of the aqueous lyophilized injection*

*(I) Preparation of an aqueous solution of aceclofenac*

On the basis of solubility data given in Table 2, the blend B was selected. The amount of individual hydrotropic agent required is less, compared to the other blends with higher solubilities.

**Table 2:** Equilibrium solubility of aceclofenac in different media

**Solvent**	**pH of solvent system**	Solubility* (g/100 mL)	**Solubility enhancement ratio**
De-mineralized (DM) water	6.5-7.2	0.018	-
30% urea solution	7-7.5	0.529	29.388
30% sodium citrate dihydrate solution	7.8-8.0	0.076	4.222
Blend A	8-8.5	1.322	73.444
Blend B	8-8.5	5.047	280.388
Blend C	8-8.5	5.082	282.333
Blend D	8.5-9	5.214	289.666
Blend E	8.7-8.9	5.733	318.500
Blend F	8.5-9.1	6.562	364.555
Blend G	8.5-9.2	7.354	408.555
Phosphate buffer	7.4	0.065	3.611
Phosphate buffer	8.0	0.069	3.833
Alkaline borate buffer	9.0	0.075	4.1665

* Average of 3 determinations

 Therefore, it was thought worthwhile, to solubilibilize aceclofenac using blend B and to formulate an aqueous injection (100 mg/2.5 mL). For the preparation of an aqueous solution of aceclofenac, about 35 mL of a hydrotropic blend (20% urea and 10% sodium citrate) solution was added into a 50 mL amber colored volumetric flask. Then, weighed amounts of aceclofenac were transferred into a volumetric flask and the flask was sonicated for a sufficient period of time until complete dissolution of drug. Next, the volume was made up to 50 mL with the same hydrotropic blend solution. In the next stage, flushing was conducted, using the nitrogen gas for 15 min. Prior to lyophilization, the initial solution was diluted twice with water for injection, for reducing the solid content per mL. This solution was stable for at least 12 h under a refrigerated condition.

*(II) Treatment of packaging material*

Amber colored glass vials of 15 mL capacity were washed several times with distilled water. All these vials were dried and sterilized by dry heat in an oven at 160°C for 2 h in inverted position. Rubber plugs were used for plugging the vials were first washed several times with distilled water and then autoclaved at 15 lbs/sq. inch (121°C) for 20 min and finally dried in a vacuum oven.

*(III) Preparation of an aseptic area*

The walls and floor of aseptic room were thoroughly cleaned and then disinfected with 5% phenol solution. Room was fumigated using 40% formaldehyde solution prepared in distilled water. Fumigation was allowed to carry out overnight. The laminar airflow bench was cleaned and wiped out with a 70% ethanol solution. UV light was switched on 30 min prior to filling the injectable solutions into vials.

*(IV) Aseptic filtration*

The aqueous solution of aceclofenac was prepared as above and sterilized by filtration under the nitrogen pressure through a 0.22 μm disposable membrane filter (Millipore), fitted in a filtration assembly, of 500 mL glass bottle. The whole assembly was sterilized by autoclaving at 15 lbs/sq. inch (121°C) for 15 min.

*(V) Final flushing with the nitrogen gas*

The sterilized aqueous solution of aceclofenac was flushed with the sterile nitrogen gas, aseptically, and 5 mL volumes were filled into vials and capped with slotted rubber plugs.

*(VI) Freezing process*

The individual vials were then placed on the condenser plate of the lyophilizer, and the temperature was allowed to reach –70°C.

*(VII) Drying process*

The vials were immediately transferred from the condenser plate to the drying plate and the sublimed water vapor was allowed to escape. The vacuum was set at 100 mTorr and the vials were allowed to dry for 72 h. After complete drying of the vials, the vacuum was released. The vials were closed and sealed by aluminium caps using a vial sealer and stored at 2-8ºC.

*Characterization of the formulated lyophilized aceclofenac injection*

*(I) Moisture uptake kinetics*

Immediately, after removal of the optimized formulation from the lyophilization chamber, the cake was transferred to an electronic balance in order to observe its weight. The cake was then exposed to atmospheric condition. The increase in weight of cake was measured for over 2 h and presented as the % of original weight. A control sample (cake in a sealed vial) was also weighed by measuring the increase in weight at room temperature (29).

% Moisture uptake = (Weight at time T – Initial weight) x 100/Initial weight

*(II) XRD studies on the formulated lyophilized injection*

Powder X-ray diffractometry patterns of the lyophilized sample and drug were obtained at room temperature using a RU-H_3_R, horizontal rotaflex rotating anode X-ray generator instrument (Rigaku International Corporation, Tokyo). The powder was spread on a graticule and pressed in such a way to prevent powder from falling while placing the grticule in a vertical position. The graticule was placed in sample holder and exposed to C_u_K_α_-radiation (40 KV, 50 MA), 2θ = 5^o^ to 40^o^ at a scanning speed of 4^0^/min and a step size of 0.02^o^ 2θ.

*(III) Study of reconstitution time*

For reconstitution of the lyophilized cake, 2.5 mL of water for injection was injected into the vial through the rubber closure. The vial was then gently swirled for proper mixing of the content. The reconstitution time was 8 sec for the developed lyophilized aceclofenac injection formulation. The reconstitution time was not affected on storage for 1 month in the refrigerator.

*(IV) Clarity testing of reconstituted injection*

Clarity test was performed by visually inspecting the externally clean vial under a good light, baffled against reflection into the eyes, and viewed against black and white background, with the content set in swirling motion.

During the performance of clarity test on the reconstituted lyophilized injection formulation, no black or white particles were seen.

*Stability studies*

*(I) Stability of aceclofenac in bulk solution*

The stability of aceclofenac in the bulk solution was studied for 12 h under room temperature and refrigerated (2 to 8˚C) conditions by the HPLC method (30).

*(II) Physical stability study of formulated lyophilized injection of aceclofenac*

The vials were subjected to physical stability studies by keeping the vials at different temperatures and humidity conditions. A control sample was kept under refrigerated condition and the lyophilized injection was observed for 30 days for color, and pH change, and appearance of any precipitation after reconstitution.

*(III) Chemical stability study*

The vials were subjected to stability studies by keeping them at different temperature and humidity conditions. A control sample was kept under refrigerated conditions. The amount of aceclofenac was estimated by a HPLC method at 15 days and 30 days time intervals and expressed in terms of the % drug remaining. The initial drug content was taken to be 100%. Zynec injection (aceclofenac injection; each mL containing aceclofenac BP and TCL-S 101 q.s., for im use only, manufactured by Themis Medicare Ltd. and Marketed by Zydus Alidac) was also similarly subjected to the stability study.

*(IV) Dilution profile of the reconstituted injection*

The lyophilized injections were reconstituted (by adding WFI) and were subjected to dilution studies (for precipitation, if any) with normal saline (0.9% NaCl) and 5% dextrose solution, as shown in Table 9. This study was performed to check the stability towards precipitate formation, when the drug is administered through an iv infusion (LVPs). This is needed, since there are chances of drug (poorly water-soluble) precipitation due to dilution of the hydrotropic agents.

## Results and Discussion

The solubility determination of aceclofenac was carried out in distilled water, hydrotropic solutions (30% urea and 30% sodium citrate) and solutions containing different concentrations of hydrotropic agents (urea and sodium citrate). The results of solubility studies are presented in Table 2. It seems from the results that the aqueous solubility of aceclofenac was increased more than 250 times in hydrotropic blends (except for blend A which was 73.44 times), 5 and 25 times in 30% sodium citrate and 30% urea, respectively. It is concluded that the solubility of aceclofenac increases synergistically by mixed hydrotropy. To find out the influence of pH on solubility of aceclofenac, the solubility of aceclofenac was determined in buffers of pH 7.4, 8.0 and 9.0 (pH range of hydrotropic blends). The solubility of aceclofenac increases slightly, with an increase in pH (maximum 4 times), which may be due to the acidic nature of aceclofenac. Thus, it can be said that, the solubility enhancement of drug by hydrotropes is not entirely due to a pH effect, but it is largely due to hydrotropy. 

**Figure 2:**
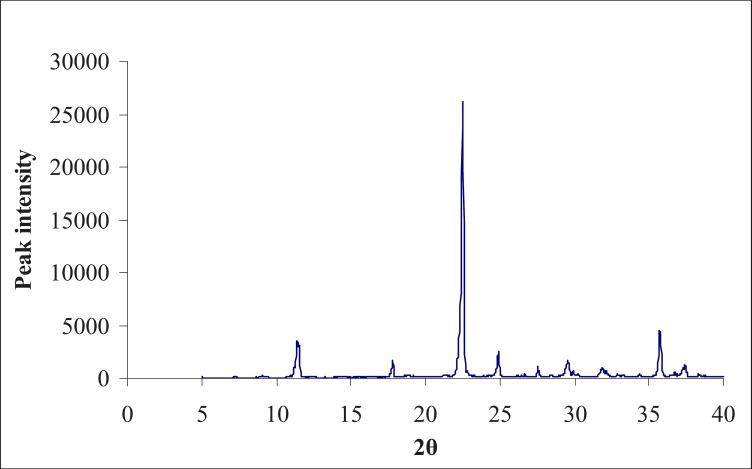
X- ray diffractogram of the physical mixture of aceclofenac and hydrotropic agents

The UV spectral data (Table 3) of aceclofenac in hydrotropic solution of urea and sodium citrate and its blend (urea + sodium citrate), showed a slight shift in λ_max_, which can be due to minor electronic changes in the structure of drug molecule. This is not indicative of any complex formation, and can be evidenced by the formation of new chromophores. 

**Table 3:** UV Spectral data analysis of the solubilized system of aceclofenac with hydrotrope solution for λ_max_

**Solubilizing agent **	**λ**_max _ **(nm) **
Aceclofenac/methanol	275.0
Aceclofenac/sodium citrate	273.0
Aceclofenac/urea	274.0
Aceclofenac/blend (urea + sodium citrate)	272.8-274.6

The results of TLC study (Table 4) revealed that there is nearly no considerable change in R_f _value of aceclofenac solubilized in methanol and aceclofenac solubilized in the hydrotropic blend solution. From the results of TLC study, it can be concluded that there is no salt or complex formation of drug with the hydrotrope molecules. 

**Table 4:** R_f_ values of aceclofenac and solubilized product

**System **	**R**_f _
Drug in methanol	0 .29
Drug in blend B (20% urea+10% sodium citrate)	0.30

The results of moisture uptake kinetics (Table 5) study of lyophilized powder clearly indicated that the lyophilized cakes are prone to moisture uptake when exposed to normal atmosphere as shown by the increase in the weight of the sample. However, there was no increase in weight of the control sample, which indicates that the sample when kept under controlled conditions, is safe and there is no moisture uptake. The amount of moisture uptake was 8.33% in 120 min, when sample was exposed to atmospheric conditions. 

**Table 5:** Moisture uptake kinetics of the lyophilized cake

Time (min)	Weight of the control sample (mg)	Weight of the exposed sample (mg)	Moisture uptake by the exposed sample (%)
0	852.1	852.6 (Initial Weight)	0
15	852.1	861.8	1.08
30	852.1	872.1	2.40
45	852.1	883.7	3.64
60	852.1	891.1	4.51
75	852.1	901.1	5.58
90	852.1	914.2	6.16
105	852.1	927.0	7.44
120	852.1	935.9	8.33

**Figure 3:**
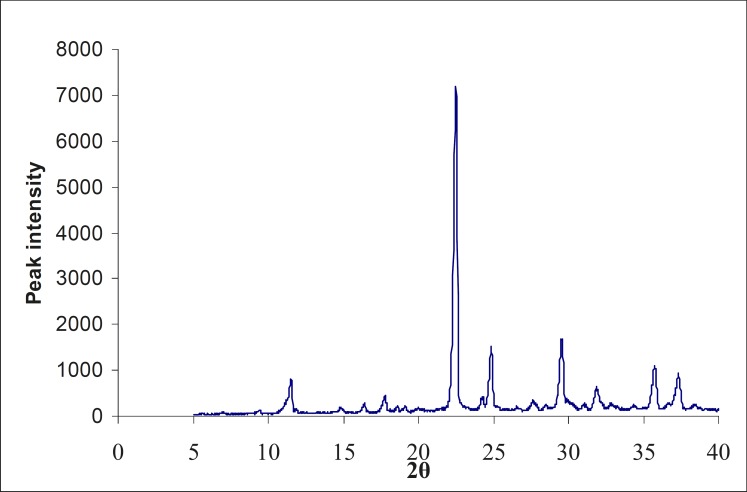
X-ray diffractogram of the formulated lyophilized injection (Formulation code: AUSC4P3)

The X-ray diffractogram of the physical mixture and lyophilized powder are shown in Figures 2 and 3. The physical mixture showed intense peaks due to crystallinity. The peaks in the lyophilized sample showed less intense peaks at the same 2θ values, due to partial conversion of drug from the crystalline form to an amorphous form. 

The reconstitution time was found to be 8 sec for the developed lyophilized aceclofenac injection. The reconstitution time was not affected by storage for 1 month in a refrigerator. 

The results of stability studies on aceclofenac in the bulk solution (Table 6), indicated that the aceclofenac is stable in the bulk solution for 4 h at room temperature and 12 h under refrigerated conditions. Thus, the same solution can be used for lyophilization within 4 h and 12 h, respectively, under unavoidable conditions. 

**Table 6:** Stability data of aceclofenac in the bulk solution at different time periods

**Time (h) **	**% Drug remaining **	
	**Refrigerated condition (2-8°C) **	**Room temperature **
0	100.00	100.00
2	100.00	99.86
4	100.00	99.67
6	99.98	96.27
8	99.95	92.54
10	99.92	91.23
12	99.91	90.01

The results of physical stability study (Table 7) showed that there is no change in physical parameters (pH, color and precipitation) after 30 days in different temperature and humidity conditions (refrigeration condition, room temperature, 40°C/75% RH and 55°C). 

**Table 7:** Physical stability of the lyophilized aceclofenac injection

**Conditions **	**Physical stability parameter **					
	**pH **		**Color **		**Precipitation **	
	**Initial **	**After 30 days **	**Initial **	**After 30 days **	**Initial **	**After 30 days **
**Refrigeration (2-8°C) **	6.51	6.54	White	White	No ppt.	No ppt.
**Room Temperature **	6.51	6.59	White	White	No ppt.	No ppt.
**40°C/75% RH **	6.51	6.64	White	White	No ppt.	No ppt.
**55°C **	6.51	7.21	White	White	No ppt.	No ppt.

**ppt.= precipitation**

The results of chemical study (Table 8) showed that the developed formulation is sufficiently stable at room temperature as a lyophilized product. The stability data of the marketed formulation at different temperatures indicated that the marketed product is less stable than the lyophilized aceclofenac injection formulation. 

**Table 8:** Stability of the developed aceclofenac injection formulation (AUSC4P3) and the marketed product

**Time (days) **	**Formulation **	**% Residual drug **		
		**Room temperature **	**40°C/75% RH **	**55°C **
**0 **	Developed formulation (AUSC4P3)	100.00	100.00	100.00
	Marketed formulation (Zynec injection)	100.00	100.00	100.00
**15 **	Developed formulation (AUSC4P3)	99.51	98.48	97.32
	Marketed formulation (Zynec injection)	99.25	97.28	96.82
**30 **	Developed formulation (AUSC4P3)	99.06	97.85	96.55
	Marketed formulation (Zynec injection)	98.78	97.82	89.23

The lyophilized injections were reconstituted (by adding WFI) and subjected to dilution studies (for precipitation, if any) with normal saline (0.9 % NaCl) and 5 % dextrose solution, as shown in Table 9. 

**Table 9:** Dilution studies on the reconstituted formulation (AUSC4P3) after dilution with the normal saline solution and 5% dextrose solution

**Dilution **	**Time (h) **													
	**Normal saline solution **								**5% dextrose solution **					
	**1 **		**2 **	**4 **	**6 **		**8 **	**24 **	**1 **	**2 **	**4 **	**6 **	**8 **	**24 **
**1:1 **	-	-		-		-	-	-	-	-	-	-	-	-
**1:5 **	-	-		-		-	-	-	-	-	-	-	-	-
**1:10 **	-	-		-		-	-	-	-	-	-	-	-	-
**1:20 **	-	-		-		-	-	-	-	-	-	-	-	+
**1:30 **	-	-		-		-	-	+	-	-	-	-	-	+
**1:40 **	-	-		-		-	-	+	-	-	-	-	-	+
**1:50 **	-	-		-		-	-	+	-	-	-	-	-	+
**1:100 **	-	-		-		-	-	+	-	-	-	-	-	+
**1:500 **	-	-		-		-	-	-	-	-	-	-	-	-

(-) No precipitation, (+) Precipitation

In case of dilution with the normal saline solution the precipitation was observed within 24 h of 1:30, 1:40, 1:50 and 1:100 dilutions (no precipitation was formed in a 1:500 dilution after 24 h, because of redissolution of precipitate). In case of dilution with the normal saline solution, precipitation was observed within 24 h at 1:20, 1:30, 1:40, 1:50 and 1:100 dilutions (there was no precipitation at 1:500 dilution after 24 h because of redissolution of the precipitate). Hence, the formulated injection could be administered through an iv infusion. 

In conclusion, the findings of this study suggest that a stable lyophilized aqueous injection of aceclofenac has been successfully developed. This study further opens the chance of preparing such lyophilized injection powders for many other poorly water-soluble drugs, using the concept of mixed-hydrotropy. 

Similar to the aceclofenac aqueous formulation, made using a combination of physiologically compatible hydrotropic agents (urea and sodium citrate), there is a good scope for other poorly water-soluble drugs to be developed as their aqueous formulation. This could be done, using a combination of suitable hydrotropic agents at reduced concentrations. The proposed hydrotropic agents are known to be safe. Hence, toxicity and safety related issues may not raise concern and would suggest their adoptability for large scale manufacturing. The proposed techniques would be economical, convenient and safe. Thus, this study opens the chance of preparing aqueous formulations of poorly-water soluble drugs, if chemical stability of the drug remains unaffected.
